# A High Variability of Mixed Infections and Recent Recombinations of Hepatitis B Virus in Laos

**DOI:** 10.1371/journal.pone.0030245

**Published:** 2012-02-22

**Authors:** Iris E. Andernach, Prapan Jutavijittum, Bounthome Samountry, Amnat Yousukh, Te Thammavong, Judith M. Hübschen, Claude P. Muller

**Affiliations:** 1 Institute of Immunology, Centre de Recherche Public-Santé/National Public Health Laboratory, Luxembourg, Luxembourg; 2 Department of Pathology, Chiang Mai University, Chiang Mai, Thailand; 3 Department of Pathology, University of Health Sciences, Vientiane, Lao PDR; 4 National Blood Transfusion Centre, Lao Red Cross, Vientiane, Lao PDR; Institute of Infectious Disease and Molecular Medicine, South Africa

## Abstract

In Lao PDR, where more than 8% of the population are chronic carriers of HBsAg, multiple genotypes and subgenotypes co-circulate and are prone to generate recombinant viruses. Phylogenetic analyses of multiple clones per donor revealed mixed infections of subgenotypes B1, B2, B4, C1, C5, I1 and I2 in almost 6% of HBsAg positive rejected blood donors. Recombination analyses and distance calculations furthermore showed that about 65% (17/26) of the mixed infected donors showed recombinations in the S-gene alone, involving the predominant genotypes B and C. These results suggest that, at least in Laos, hepatitis B virus (HBV) mixed infections lead to frequent recombinations. In many donors with recombinant strains, the recombinant fragment and a non-recombinant strain of the same genotype co-existed (127/185 analysed recombinant fragments). For a large proportion of these (60/127), the most closely related known virus was found, although not always exclusively, in the same donor. Recombinant virus strains are largely distinct. This is reflected in an unexpected diversity in recombination breakpoints and the relatively rare recombinations with identical recombination patterns of the same genotypes in different donors. Recent recombination events would explain the limited spread of each of the recombinants. Using a published mutation rate of 4.2×10^−5^ mutations per site and year, the observed minimum genetic distances of 0–0.60% between parent strain and recombinant fragment would correspond to 0–71 years of evolution from a most recent common ancestor (MRCA). Thus several lines of evidence are suggestive of recent independent recombination events, a proportion of these even occurring within the same donors. In conclusion, our analyses revealed a high variability of mixed infections as a very probable breeding ground of multiple variable recombination events in Laos that so far have not led to new dominant strains.

## Introduction

Hepatitis B virus (HBV), a major global public health burden, is classified into 8 recognised genotypes A-H [1], [2] and a tentative genotype I found in Laos [3], Vietnam [4], [5], [6] and also in Northwestern China [7] and India [8]. Recently a tenth genotype J has been proposed in a Japanese patient [9]. With the exception of genotypes E, G and H, HBV genotypes are divided into subgenotypes with more or less distinct geographic distributions [1], [2], [10], [11].

In Asia, genotypes B and C largely dominate and are divided into a number of subgenotypes. While B1 (formerly Bj) is mainly found in Japan and represents the non-recombined B subgenotype [12], subgenotypes B2 to B4 (formerly Ba) largely originate from mainland Asia and are recombinants with genotype C. B2 is mainly found in China and Southeast Asia, B3 mainly in Indonesia and B4 in Laos, Vietnam, Cambodia and China [2], [3], [13]. The subgenotypes B5 and B6 have been isolated in the Philippines and from the Canadian Inuit populations, respectively, and subgenotypes B7, B8 and B9 have been proposed in Indonesia [14], [15], [16]. Also genotype C strains group into several subgenotypes. According to the nomenclature from Huy et al. [11], subgenotype C1 represents predominantly strains from mainland Asia (Laos, Vietnam, Thailand and Myanmar), while C2 is prevalent in Japan, Hong Kong, China and Korea [2], [10], [11], [17]. Subgenotype C3 consists of strains from Oceania, C4 of strains exclusively from Australia and subgenotype C5 strains have been reported from the Philippines, Indonesia, Vietnam and Laos [3], [4], [18], [19]. Additionally, subgenotypes C6 to C10 have been proposed recently for strains from the Philippines and Indonesia [3], [19], [20].

In highly endemic countries, particularly in Asia where different HBV genotypes and subgenotypes co-circulate, mixed infections [21], [22], [23], [24] and recombinations have been described [3], [25], [26], [27], [28], [29], [30], [31]. For instance, in China almost 50% of infections are mixed [22] and mixed infections have also been reported from Thailand [32].

Recombination events do not seem to be negligible artefacts of HBV co-infections, as recombinant strains have become the dominant variant in certain regions. For example subgenotype Ba, a B/C recombinant, prevails in major parts of mainland Asia, and a recombinant between genotypes C and D has become the predominant variant in Tibet [27]. Furthermore, recently a recombinant variant that circulates in Laos and Vietnam has been proposed as a new HBV genotype I [3], [4], [5], [6]. Nevertheless, only few systematic studies of HBV recombinants have been published from regions with intensive co-circulation of several HBV subgenotypes [3], [12], [29].

In Laos, with 8.7% of chronic carriers [33], multiple HBV sub-/genotypes co-circulate [3] and were shown to belong to genotype C (55.4%), with subgenotypes C1 (93.1% of genotype C strains), C5 (6.4%) and C3 (0.5%), genotype B (42.2%), with subgenotypes B4 (78.5% of genotype B strains), B2 (11%), B5 (9.8%) and B3 (0.6%) and the suggested genotype I (2.4%), with subgenotypes I1 and I2 [3]. As a result of the high prevalence of chronic HBV carriers and the co-circulation of multiple genotypes and subgenotypes, Laos is prone to generate recombinant viruses. Here, we systematically analysed mixed infections and recombinations in 42 HBV strains from HBsAg positive first time blood donors. Our study revealed an unusual cauldron of mixed infections as a breeding ground of multiple and highly variable recombination events that so far have not led to new dominant strains.

## Methods

### Ethics Statement

Blood samples were collected by the National Blood Transfusion Centre, Lao Red Cross, Lao PDR from voluntary blood donors. All blood donors gave written consent to test their blood for Hepatitis B and C virus, HIV and syphilis and to use the leftover of the samples positive for HBsAg for the present study. The study has been approved by the competent Ethical Committee of the Faculty of Medical Sciences, National University of Laos and was conducted according to national and international guidelines.

### Specimens

DNA was extracted using the QIAGEN DNA Blood Mini Kit (QIAGEN, Venlo, The Netherlands), from sera of 40 HBsAg positive, but otherwise healthy, rejected first time blood donors from Vientiane City and Central provinces of Laos (collected in 2004/2005) and of 2 donors from the North of Laos (2006).

### Amplification and cloning

The HBV S gene was amplified in a semi-nested format as described previously [34], using forward primer P2f (5′-CCTGCTGGTGGCTCCAGTTC-3′) and reverse primer 979 (5′-CAAAAGACCCACAATTCTTTGACATACTTTCCAAT-3′). PCR products were cloned, using the TOPO TA cloning kit (Invitrogen, Leek, The Netherlands). Bacteria were plated onto Luria-Bertani-Broth agar plates, in the presence of kanamycin [30 µg/ml] and X-Gal. White colonies were picked and inserts were amplified by colony-PCR using M13fw (5′-GTAAAACGACGGCCAG-3′) and M13rv (5′-CAGGAAACAGCTATGAC-3′) primers.

### Sequencing and phylogenetic analysis

The M13 PCR products, with 10 to 31 clones per donor, were purified and sequenced as described before [34] with above primers. Phylogenetic analysis and distance calculations were performed, using the MEGA v.4 software [35] with the neighbour-joining method of the Kimura 2-parameter model and a γ value of 0.6, with 1,000 bootstrap replicates. Sequences were submitted to EMBL/GenBank/DDBJ under accession numbers: HE652134 - HE652863.

### Recombination analysis

Bootscan analyses were performed using SimPlot v.3.5.1 [36] with a window size of 200 bp and a step size of 20 bp. Recombination breakpoints were confirmed by comparing each recombinant sequence with consensus sequences with a threshold of 50% for inclusion in the consensus nucleotide sequence (consensus-50) [25] of subgenotypes B1-B8, C1-C7 and I1, I2. Of these, the subgenotype B6 consensus-50 harboured one ambiguous nucleotide. Additionally, consensus-50 sequences of genotypes B, C and I were included in the analysis, reconstructed from the associated consensus-50 sequences of the above subgenotypes to exclude a bias of the differently sized sequence sets available. The individual fragments of the potential recombinant strains were confirmed by phylogenetic analyses.

## Results

### Genotypes and subgenotypes

In 9157 blood donors in Vientiane city and central provinces from 2004 and 2005 the HBsAg prevalence was 8.8%. Of the 498 available samples of HBsAg positive blood donors, 453 were found to be PCR positive, 446 of them at least for the S gene. Sequence analyses of these donors revealed at least 5 ambiguous nucleotides within the amplified S gene of 40 donors, indicative of infections with several HBV variants. In addition, 2 donors from Luang Prabang in the North of Laos were suspected to be mixed infected and included in the analyses. The S gene PCR products from these 42 donors were further characterized by extensive cloning. Phylogenetic analyses revealed that among the 730 clones of these 42 donors, 16 donors had only clones of the same subgenotype (Table 1). Of these 16 non-mixed/non-recombined samples, strains from 12 donors belonged to subgenotypes B4 and one each to C1, and I1. Genotype B strains of the remaining 2 donors could not further be subgenotyped (Table 2; nomenclature according to Huy et al. [11]). The Asian recombinants, assigned to subgenotype B2-B4 (former Ba), and the proposed genotype I are not considered recombinants for the purpose of this study.

**Table 1:** Number of donors suspected to be mixed infected with at least 2 sub-/genotypes of HBV, Lao PDR.

Samples	No. donors
Detection-PCR positive	453
S-gene PCR positive	446
Total analyzed/suspected mixed infection	42
Non-mixed, non-recombinant	16
Total mixed	26
Mixed, non-recombinant	9
Mixed, recombinant	17

**Table 2:** Sub-/genotypes in HBV mixed infections in Lao PDR.

Category	Sub-/genotype(s)	No. donors
Donors suspected/analyzed	n/a*	42
Non-mixed, non-recombinant donors (n = 16)	B	2
	B4	12
	C1	1
	I1	1
Clones in mixed, non-recombinant donors (n = 9)	B4, B	1
	B4, B2	1
	B4, C1	1
	C1, B	2
	C1, C5	1
	C1, I2	1
	B1, B4, C1	1
	B4, C1, C5	1
Clones in mixed, recombinant donors (n = 17)	B	1
	B4, B, C1	2
	C1, C5, B4	1
	B4, C1	11
	C1, C	1
	C1, I1	1

*n/a: not applicable.

#### Mixed, non recombinant

The other 26 donors, or 5.8% of the 446 donors that were PCR positive for the S gene, were mixed infected with different variants. For 9 of these donors (14 to 30 clones) all clones were clearly assigned to a sub-/genotype, whereas the remaining 17 donors (3.8% of the 446 S gene PCR positive donors) had at least one (and up to 27 clones of the 10 to 31 clones generated) with signs of a recombination within the S fragment (Table 1). In addition, recombination events were detected in 2 strains from Laos already published on NCBI (accession nos. FJ023979, FJ023832) which were included in the analyses.

In the above 9 donors with mixed, non-recombined variants, at least one clone of 7 of the donors was assigned to genotype C1, while the second sub-/genotype varied and belonged to subgenotypes B4 (n = 1 donor), C5 (n = 1 donor), I2 (n = 1 donor), and non-subgenotypable genotype B clones (n = 2 donors), while for one donor each B1 and B4, or C5 and B4 was found in addition to C1. For two donors no clone was assigned to genotype C and clones were attributed to genotypes B2 and B4 as well as B4 and non-subgenotypable B clones (Table 2).

#### Mixed, recombinant

**Figure 1:**
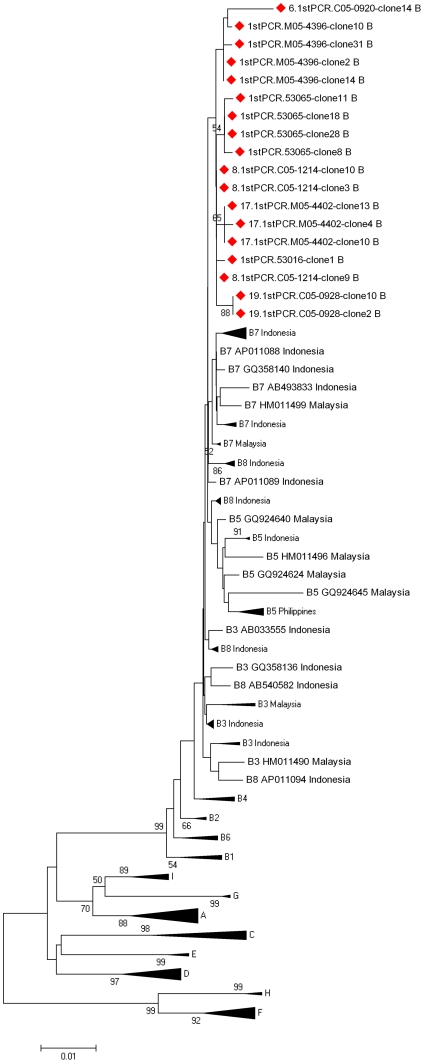
Phylogenetic clustering of selected HBV/B subgenotype B3, B5, B7 and B8 strains from GenBank and selected genotype B strains from Laos. Non-subgenotypable clones are indicated by red diamonds. Scale bar indicates nucleotide substitutions per site.

In the other 17 donors with mixed-infections at least one clone showed signs of recombinations, and at least one clone showed no such evidence. 16 of these donors had non-recombined clones, that were assigned to genotype C, 15 to genotype B and 1 to genotype I, with subgenotypes C1 (n = 16), C5 (n = 1), B4 (n = 14) and I1 (n = 1) (Table 2). Strains of one donor were attributed to genotype C, but clustered separately from all HBV/C subgenotypes. Additionally, genotype B clones from 3 donors could not be subgenotyped, as the reference strains for genotype B subgenotypes B3, B5, B7 and B8 clustered interspersed with each other and the investigated strains (Figure 1).

#### Deletions and insertions

The S gene of the 730 clones was 681nt long, except for 7 clones, consisting of genotypes C1, B4 and B which contained single nt deletions (3 clones in donors with mixed infected, non-recombinant clones and 4 clones in donors with recombinant strains). For another 6 clones, consisting of genotypes B4 and B, the S gene sequence could not be fully recovered.

### Control experiments

Considering the large proportion of recombinant strains detected in this study, we performed extensive tests to exclude that there may be recombinants due to PCR or other artefacts. Therefore, M13 products of cloned, characterized HBV/B and HBV/C strains were mixed in various ratios (1∶25, 1∶5, 1∶1, 5∶1 and 25∶1) and concentrations (1 ng and 100 ng total DNA) and amplified using different elongation times (1 min elongation as in the study protocol and a reduced elongation time of 20 sec). PCR products were cloned by TOPO TA, using the above standard protocol, amplified by M13 PCR and sequenced. In the 1 min elongation PCR only rare recombinants (<2%, 2/107 clones) were found (Table 3). Only when the elongation time was reduced to 20 sec the rate of recombinations increased to an overall 16% (18/111 clones). At 1 ng final concentration and 20 sec elongation 6.4% of clones showed recombinations. This increased to 39.4% when using 100 ng template DNA *and* the short elongation time (Table 3). Thus recombinant artefacts seem to depend on high template concentrations *and* short elongation times.

**Table 3:** Number of PCR-induced recombinant clones in control experiments.

	Template concentration	
Elongation time	1 ng	100 ng
20 s	6.41% (5/78 clones)*	39.39 (13/33 clones)
60 s	2.53% (2/79 clones)	0% (0/28 clones)

*Clones for each condition originate from HBV/B and HBV/C strains mixed for amplification in ratios 1∶25, 1∶5, 1∶1, 5∶1 and 25∶1.

After PCR amplification of our clinical samples the highest final concentration of PCR product before cloning was 154 ng/ul, corresponding to a template starting concentration considerably lower than the nanomolar concentrations of DNA analysed in the above control experiments. Although we cannot exclude that some recombinants may have developed only when the concentration of template increased during the later amplification cycles, these recombinants would be relatively rare and unlikely to be picked up during the cloning. This confirms that under our experimental conditions only very few recombinants (of the observed 9.9% of recombinant clones) would be the result of PCR artefacts. We also excluded that recombinations may have occurred during cloning. Thus, the observed high prevalence of recombinant clones in 65.4% of mixed infected donors is essentially free of PCR dependent or other artefacts.

### Distance calculations of non-mixed, non-recombined samples

Distance calculations were performed in clearly subgenotypable clones of individual donors, with at least 5 clones of the respective subgenotype, including only clones for which the full S-gene could be recovered. These clones formed quasispecies within individual donors and revealed relatively low mean intra-group diversities for subgenotypes B2 (0.47%; n = 1 donor) and B4 (0.08–0.47%; n = 24), subgenotypes C1 (0.05–0.82%; n = 11) and C5 (0.04%; n = 1), as well as subgenotypes I1 (0.34%; n = 1) and I2 (0.25%; n = 1).

### Amino acid analysis of clearly genotypable strains

The comparison of 651 clearly genotypable strains (excluding the 7 clones with nucleotide insertions that were not considered for analyses on the amino acid level) with consensus-50 sequences of genotypes B, C and I as well as their subgenotypes revealed multiple and variable amino acid (AA) changes over the S protein.

#### HBV/B clones

In 469 HBV/B clones, including the 6 clones of which the S gene sequence could not be fully recovered, AA substitutions occurred in 120 of 227 AA positions. In 7 of these positions substitution occurred in >5 clones: T27A/I/V (n = 7 clones of d = 5 donors), S61L/A (n = 6, d = 6), P62L (n = 6, d = 5), C76Y/R (n = 8, d = 6), P120S/T/L (n = 20, d = 14), M133I (n = 31, d = 2), T140I (n = 8, d = 6). Furthermore, a total of 6 internal stop codons (*) were detected.

#### HBV/C clones

In 154 genotype C clones we found AA substitutions in 56/227 positions, with 2 occurring in >5 clones: I92T (n = 11, d = 1), T118M/P (n = 51, d = 15). In the genotype C strains, a total of 4 internal stop codons were detected.

#### HBV/I clones

In the 28 genotype I clones, 19/227 AA positions were found to harbour substitutions. Due to a lower sample size of HBV/I strains, only 2 AA positions with >1 substitutions per site were further characterized. The investigated strains were found to harbour P111L/Q (n = 2, d = 2), I226N (n = 12, d = 1). In the genotype I strains, a total of 2 internal stop codons were detected.

#### Vaccine or treatment associated mutations

Despite the frequent amino acid substitutions only rare mutants had been described previously as vaccine or treatment associated mutations [37], [38], [39], including P120T (n = 5, d = 4), G145R (n = 1), W172* (n = 2, d = 2), W182* (n = 2, d = 2), L192F (n = 1), W196* (n = 1), M198I (n = 18, d = 2) and W199* (n = 1).

#### HBsAg subtypes

All 4 major HBsAg subtypes, that have been described previously in genotype B and C strains from Asia [2], were found among the analysed clones. In the 469 analysed genotype B strains HBsAg subtype ayw1 predominated (n = 444 clones, d = 35 donors), while 18 clones (d = 3) were attributed to adw2 and one (d = 1) to ayw2. Three clones (d = 3) harboured 127S and were attributed to ayw. For three additional clones no HBsAg subtyping could be performed, as two clones harboured AA substitutions 122G or 160E and for one clone the HBsAg sequence could not be fully recovered.

The 154 genotype C strains belonged primarily to subtype adr (n = 122, d = 25), but also to adw2 (n = 30, d = 4), ayr (n = 1, d = 1) and ayw1 (n = 1, d = 1).

In the 28 genotype I strains on the other hand both adw2 (n = 16, d = 2) and ayw1 (n = 12, d = 1) prevailed.

### Description of recombinant clones

**Figure 2:**
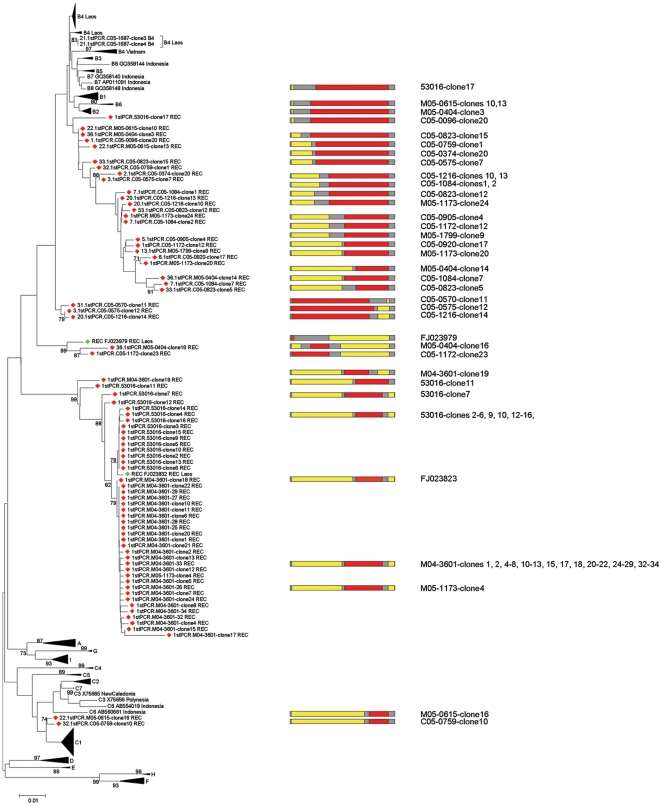
Phylogenetic clustering and recombination patterns of recombinant strains. Colour codes indicate genotypes B and C (red and yellow) involved in the recombination and regions homologous between at least 2 genotypes (grey). Recombinant strains from this study (red) or detected in previously published strains (green) are indicated by diamonds.

A total of 72 recombinant clones were detected in the 42 analysed donors, from which 730 clones were generated. In 17 donors at least one recombinant clone was detected. All of the 72 recombinant clones exhibited one or two recombination site(s) within the S gene and revealed recombinations between genotypes B and C (Figure 2). Of the 185 genotypable fragments 72 were assigned to genotype B and 113 to genotype C.

In none of the analysed recombinant strains the recombination breakpoint could be exactly defined because homologous stretches of at least 17 nt length separated the recombined fragments (Figure 2). Interestingly, recombinations occurred all over the S-gene: nt 1–170 with 10 recombinant sites, nt 171–510 with 58 recombinant sites and nt 511–681 with 45 recombinant sites (Figure 2).

For each of the individual 185 fragments of the 72 cloned recombinants the most similar strains were identified by phylogenetic analyses and pairwise comparison with a carefully selected dataset that was composed of all available, clearly subgenotypable full-length genome strains from NCBI (accessed 08/2011) and all non-recombinant strains from this study.

Based on the simplest evolutionary model with a mutation rate of 4.2×10^−5^ mutations per site and year [40], the time of evolution from a hypothetical most recent common ancestor (tMRCA) was calculated. For 127 of the 185 fragments a non-recombinant strain of the same genotype was detected in the same donor. Two of the 127 fragments were most closely related (Kimura-2-parameter, pair wise genetic distances) to strains of the same donor (0% and 0.5%), with minimum genetic distances corresponding to 0 and 60 years of evolution from a hypothetical MRCA, while 58 fragments (of the 127) were genetically equidistant to clones found within the same donor and to strains from other origins (0–0.7%, 0–83 years to a MRCA). In 13 of the latter 2 and 58 fragments, the tMRCA was lower than or equal to the age of the donor. The remaining 67 of the above 127 fragments were most closely related to strains from another source (0–7.6%, 0–905 years to a MRCA).

58 of the above 185 fragments of recombinant strains, however, originated from donors that were *not* coinfected with a strain of the genotype found in the recombinant fragments. Of the latter 58 fragments 1 fragment was found to be most similar to a strain from Laos (0%, 0 years to a MRCA), and 56 fragments were equidistant to strains within and outside Laos (0–7.6%, 0–905 years to a MRCA). 1 fragment was most similar to strains outside of Laos (3.2%, 381 years to a MRCA).

In addition, recombination events were detected in the previously published strains FJ023979 and FJ023832 and the individual fragments (n = 5) of these two recombinant strains were attributed to genotype B and C. For 1 of these fragments the most closely related strain was found within Laos (1%, 119 years to a MRCA), while 3 fragments were equidistant to strains within and outside Laos (0–7.6%, 0–905 years to a MRCA) and 1 fragment was most closely related to strains outside of Laos (0%, 0 years to a MRCA).

### Amino acid sequence analysis of recombinant clones

Comparison of the 74 recombinant clones (including FJ023979, FJ023832) with the consensus-50 alignment revealed several consistent amino acid (AA) substitutions over the HBsAg. In these clones AA substitutions occurred in 47 of 227 AA positions. In 10 of these positions substitution occurred in >5 clones: P11H (n = 44 clones of d = 3 donors and FJ023832), T/A45P (n = 43, d = 3 and FJ023832), P/L49R (n = 43, d = 3 and FJ023832), C76F/S (n = 43, d = 3 and FJ023832), T118A/M (n = 17, d = 5 and FJ023832), F134I (n = 42, d = 3 and FJ023832), V190A (n = 27, d = 2), I218L (n = 41, d = 3 and FJ023832), F219S (n = 41, d = 3 and FJ023832), C221F (n = 41, d = 3 and FJ023832). Furthermore, 3 recombinant clones revealed internal stop codons. Of the observed amino acid substitutions only W182* had been described as vaccine or treatment induced mutation [37], [38], [39].

Similarly to the clear genotypable strains, the 4 major HBsAg subtypes [2] were found within the recombinant clones. For 16 clones as well as FJ023832 and FJ023832 the end or the beginning of a recombinant fragment fell within the ‘a’-determinant. For these clones the HBsAg subtypes were found to be ayw1 (n = 14, incl. FJ023832), adw1/2 (n = 3) and adr (FJ023979). The remaining clones were attributed to ayw1 (n = 52) and adr (n = 4).

## Discussion

### Genotypes

The 42 HBsAg positive donors from which clones were derived, were infected with a large variety of subgenotypes (B1, B2, B4, C1, C5, I1, I2) (Table 2) similar to the one observed in our earlier study [3]. Both genotypes B and C seemed to circulate with high prevalences in the investigated cohort and were found in 85.7% and 57.1% of investigated donors, with subgenotypes B4 (73.8% of all donors) and C1 (57.1%) dominating. Subgenotyping of a number of genotype B clones was, however, hampered by the mis-assignment of subgenotypes B3, B5, B7 and B8 strains currently available on GenBank (Figure 1). The low phylogenetic support and genetic distances, sometimes below 3% (between B3 and B7) [14], may require a later reclassification of genotype B strains.

The proposed genotype I, reported from several locations in Laos and from Vietnam [3], [4], [5], [6] shows evidence of recombinations between genotypes C, G and perhaps A (by SimPlot analyses), but is for the purpose of this study not considered a recombination. While genotype I had been proposed on the basis of at least 7.8% mean genetic distance to established genotypes [3], its classification as a genotype remains an open but academic debate [41] until more detailed guidelines for nomenclature and the definition of recombinants are established.

As a result of the co-circulation of HBV subgenotypes in Laos, 5.8% (n = 26) of the 446 analysable HBsAg positive blood donors, were mixed infected with multiple HBV variants (Table 1). 17 of these (3.8% of the 446 donors or 65.4% of the 26 mixed infected donors) revealed at least one recombinant strain, all originating from genotypes B and C (subgenotype C1).

The proposed genotype I, however, was relatively rare (3/42 donors). The most prominent Asian recombinants with B and C parent sequences (subgenotypes B2-B4, formerly Ba) were found in Laos in 31/42 donors, while the C/D recombinant predominant in Tibet [27] was absent. These latter widespread recombinant strains (B2-B4, I1, I2) seem to have long evolutionary histories in Asia, originating from either single or multiple very similar recombination events in a distant past, and are here no longer considered recombinants. However, these subgenotypes of B recombined further in Laos with HBV/C, most often C1, strains, to form the relatively recent recombinants described in this study.

### Recombinations

A surprisingly large number of 72 recombinants was found in 64.5% of mixed infected donors, representing 9.9% of all cloned strains. These strains largely reflect recombinant strains circulating in Laos and cannot be explained by PCR artefacts, as confirmed by our control experiments.

127 of the genotypable 185 fragments belonged to the same genotype as the co-infecting, non-recombinant strains. For 60 of these, the most closely related known virus was found, although not necessarily exclusively, in the same donor. Using a published mutation rate of 4.2×10^−5^ mutations per site and year [40], the observed minimum genetic distances of 0–0.7% between parent strain and recombinant fragment would correspond to 0–83 years of evolution from a MRCA. For 13 of these fragments (all originating from different cloned recombinants) even a lower or the same time of evolution than the age of the donor was calculated. Although, some donors with recombinant strains were not co-infected with similar (non-recombinant) parent strains, the above observations are suggestive of recent recombination events and even within the individual donors. These putative recent recombination events would explain the very limited spread of each of the recombinants and that the recombinant virus strains are largely distinct from each other (Figure 2). Only the proposed genotype I and subgenotypes B2 and B4 represent older recombinants in this region. The large number of apparently independent recent recombination events is also reflected in an unexpected diversity in recombination breakpoints (Figure 2), the locations of which were highly variable throughout the S gene. This variability in recombination sites was surprising, since earlier studies [25], [28], [30], [31] reported recombination breakpoints mostly near gene boundaries. Interestingly, all recombined fragments, independently of their breakpoints within the S gene, were separated by homologous stretches of at least 17 nucleotides, suggesting that this may be conducive to recombinations. The breakpoint diversity in these recombinants also seems to be in contrast to the dominant recombinants found in the rest of mainland Asia [2], [3], [13], [27].

When comparing the breakpoints of individual recombinant clones more closely, clones with different recombination patterns were not only found in different donors, but also within the same donor (e.g. donor M05-1173) and in similar or different strains from different donors (Figure 2). The relatively rare recombinations with identical recombination patterns of the same genotypes in different donors seem to further suggest their recent emergence, probably within the last century. Thus, several of the above lines of evidence, as well as our control experiments, suggest that many of the recombinants described here have occurred in the same donor.

Surprisingly, amino acid substitutions were found in a large proportion of investigated clones. However, these were largely free of vaccine or treatment induced mutations. They furthermore agreed largely with the HBsAg subtypes that had been described previously in genotype B and C strains from Asia [2], indicating that recombination between genotypes B and C is not adding to the diversity of HBsAg subtypes in HBV strains circulating in Laos.

Perinatal transmission is considered by some authors the most common route of infection in Asia [42]. Furthermore, superinfections are considered to be rare and the ongoing immune response and the replicative space may limit superinfections and acceleration of chronic HBV even in regions of high endemicity [43]. Since furthermore quasispecies found within the individual donors are unlikely to all be transmitted independently, one could argue that all quasispecies would emerge from the initial pool of viruses infecting at birth. Assuming further, that all quasispecies develop from a single virus, we calculated the mutation rate, required for the most distant quasispecies (of the same subgenotype) to develop since birth of the donors. The median mutation rate was found to be 1.58×10^−4^ mutations per site and year, irrespective of whether samples were included for which at least 5 or 10 clones were available. This is well within the range of published short-term mutation rates (range 1.4×10^−5^ to 7.9×10^−4^) [44]. These estimates of short-term evolutionary rates of HBV are, however, distinct from the much slower mutation rates (∼10^−9^) recently observed during co-evolution of HBV genomes in birds over several millions of years [45]. The dramatic differences between long and short-term mutation rates reflect the different evolutionary constraints during co-evolution with the host species and rapid adaption under the pressure of the host immune system. In addition, mutational saturation could be limiting and high mutation rates would not be reflected in the long-term evolution of a virus.

On the basis of the mutation rate 1.58×10^−4^ mutations per site and year, calculated above, the tMRCA would be reduced by a factor of 3.8, largely corresponding to about 0–22 years of evolution for those recombinant fragments for which the most closely related known virus was found within the same donor, further suggesting that these recombinations occurred during the lifetime of the donor. Even under the more likely assumption that more than a single virus quasispecies was infectious at birth, the above contention seems to suggest that the number of quasispecies transmitted vertically is limited.

In conclusion, in Laos, multiple HBV sub-/genotypes co-circulate, generating highly variable recent recombinants with unique breakpoints, few of which seem to have spread within the population. About 65% of mixed infected donors showed recent recombinations in the S-gene alone, strongly suggesting that, at least in Laos, mixed infections invariably lead to recombinations.
